# Mercury in *Macrolepiota procera* (Scop.) Singer and Its Underlying Substrate—Environmental and Health Risks Assessment

**DOI:** 10.3390/jof7090772

**Published:** 2021-09-18

**Authors:** Ivona Jančo, Marek Šnirc, Martin Hauptvogl, Lenka Demková, Hana Franková, Vladimír Kunca, Tomáš Lošák, Július Árvay

**Affiliations:** 1 Department of Chemistry, Faculty of Biotechnology and Food Sciences, Slovak University of Agriculture in Nitra, 949 76 Nitra, Slovakia; marek.snirc@uniag.sk (M.Š.); xchrkavah@uniag.sk (H.F.); julius.arvay@gmail.com (J.Á.); 2 Faculty of European Studies and Regional Development, Institute of Environmental Management, Slovak University of Agriculture in Nitra, 949 76 Nitra, Slovakia; martin.hauptvogl@uniag.sk; 3 Department of Ecology, Faculty of Humanities and Natural Sciences, University of Prešov, 081 16 Prešov, Slovakia; lenka.demkova@unipo.sk; 4 Department of Applied Ecology, Faculty of Ecology and Environmental Sciences, Technical University in Zvolen, 960 01 Zvolen, Slovakia; kunca@tuzvo.sk; 5 Department of Environmentalistics and Natural Resources, Faculty of Regional Development and International Studies, Mendel University in Brno, 613 00 Brno, Czech Republic; tomas.losak@mendelu.cz

**Keywords:** *Macrolepiota procera*, mercury, bioaccumulation, contamination

## Abstract

Wild-growing edible mushrooms are valuable food with a high content of proteins, fibers, antioxidants, and they are characterized by their specific taste and flavor. However, from an ecotoxicological point of view, they are a risk commodity because of their extremely high bioaccumulative capacity to accumulate the risk elements and contaminants from the environment. In the present study, we examined mercury (Hg) contamination in 230 fruiting bodies of *Macrolepiota procera* (Scop.) Singer and 230 soil/substrate samples, which were collected in foraging seasons 2015–2019 from 22 different locations in Slovakia. Total mercury content was determined by cold-vapor AAS analyzer AMA 254. The level of contamination and environmental risks were assessed by contamination factor (C_f_), index of geoaccumulation (I_geo_), and potential environmental risk index (PER). Bioaccumulation factor (BAF) was calculated for individual anatomical parts of *M. procera*. Mercury content in the soil/substrate samples varied between 0.02 and 0.89 mg kg^−1^ DW, and in mushroom samples between 0.03 and 2.83 mg kg^−1^ DW (stems), and between 0.04 and 6.29 mg kg^−1^ DW (caps). The obtained results were compared with the provisional tolerable weekly intake for Hg defined by WHO to determine a health risk resulting from regular and long-term consumption of *M. procera*.

## 1. Introduction

Nowadays, mushrooms are considered valuable foods, not only because of their attractive sensory characteristics and their culinary features but also for their nutritional properties [1]. From an environmental point of view, mushrooms have a positive impact on increasing soil fertility through their ability to break down and dissolve complex compounds into simple ones, as well as the ability to reduce or eliminate environmental pollutants [2,3]. Mushrooms (mainly ectomycorrhizal macrofungi) can accumulate high amounts of potentially harmful elements, especially when collected from heavily contaminated regions (mining sites, industrial areas) or soils with high metal content [4,5]. Mushrooms grown in such conditions are toxic and non-edible [6,7]. 

The accumulation of risk elements by mushrooms is a complex process affected by both environmental and intrinsic factors [8]. The mycelia of mushrooms spread over large areas (several square meters) and their relationships with soil and dead organic matter, as well as their symbiotic relationship with plant roots, allow an intensive exchange with substrates. Risk element content in many mushroom species correlates with the high metal content in the soil they grow in. Some authors report higher metal contents in immature fruiting bodies than in adult ones, which is explained by the transport of metal ions from the mycelium to the fruiting body occurring predominantly at the beginning of the fructification [9]. There is growing evidence that mushrooms can be suitable for biogeochemical prospecting for minerals as well as indicators of risk elements and radioactive contaminants in the terrestrial environment. Knowledge of accumulation dynamics and distribution of elements in fruiting bodies, from emergence to senescence, is essential as is standardization when choosing mushroom species as potential bioindicators and for monitoring purposes [10]. The morphological differences for different taxa in their early development would strongly suggest implications to physiology and therefore to the distribution of elements. Furthermore, fruiting body expansion may be accomplished both by cell division and, more frequently, by the expansion of existing cells which is typically achieved through rapid water uptake. Therefore, the existing variety of primordial growth may partly explain the unpredictable pattern of elemental distribution reported in the literature. The metallic and metalloid elements accumulated by the ectomycorrhizal fungi mycelium are thought to be taken up passively or actively from soil solution and substrate. Once absorbed, they are used by the mycelium and partly supplied to the ectomycorrhizal symbiont(s). Knowledge of the characteristics of minerals accumulated by fungi helps in understanding ecosystem functioning and contributes to an explanation of the nutritional value of edible species [11].

*Macrolepiota procera*, known under the common name Parasol Mushroom, formerly called *Lepiota procera*, prefers light and warm places, especially calcareous and sandy soils that are well-drained in forests, meadows, and gardens [12,13]. It is a soil-inhabiting saprophytic species growing alone, scattered in woods, at the edges of woods, or in pastures [12]. It is popular in Europe, and its cap is edible and tasty [12]. Parasol Mushroom has low-fat content and high content of proteins and minerals [14]. The species, like certain other macromycetes, is efficient in accumulating toxic mercury (Hg), cadmium (Cd), lead (Pb), silver (Ag), and some micronutrients in fruiting bodies. Due to its bioaccumulation ability, many researchers investigate metalloids and toxic metals contents in the fruiting bodies of *Macrolepiota* species commonly collected by locals for their essential micro- and macroelements [12].

Mercury, unlike other risk metals, can remain in the atmosphere and soil for the long-term and it can be distributed or migrate over long distances [15]. Sources of mercury pollution in soil include atmospheric deposition, sewage irrigation, livestock manure, and discarded mercury-containing appliances [16]. Inorganic mercury in the environment can be converted to highly neurotoxic methyl mercury (MeHg) [17]. Mercury ranks among the most frequently determined elements in mushrooms. *M. procera* and some other species are mercury bioaccumulators [18]. After entering the human body, mercury exerts a variety of adverse effects on human body organs, e.g., kidney, liver, respiratory system, skin, nervous systems and immune system, reproductive and developmental defects, genotoxicities, and cardiovascular disorders [19].

The present study aims to determine the level of mercury contamination of *M. procera* and soil/substrate samples. Ecological risks of Hg were evaluated by calculating contamination factor, index of geoaccumulation, potential environmental risk index, as well as the bioaccumulation factor. Third, to assess the health risk resulting from the consumption of the studied mushroom by applying provisional tolerable weekly intake.

## 2. Materials and Methods

### 2.1. Statistical Analysis and Data Processing

Table 1 summarizes the GPS coordinates of the sampling areas and the number of samples. The data were processed in the open-source QGIS software (v3.10; multilevel B-spline interpolation method).

### 2.2. Study Areas, Sampling, and Sample Preparation

**Figure 1:**
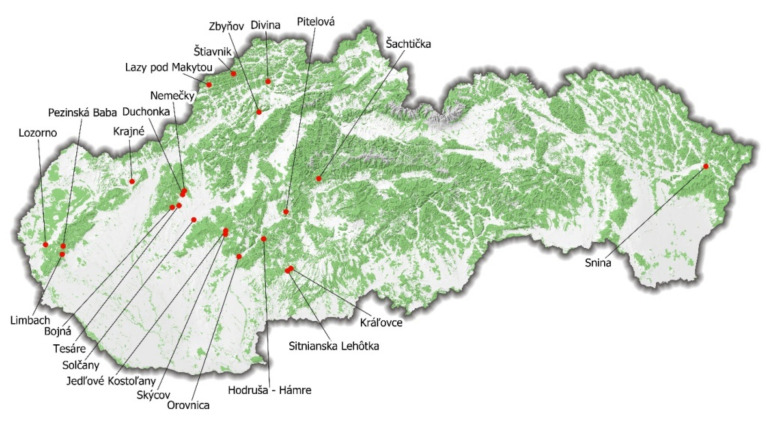
Mushroom and its underlying soil sampling localities in Slovakia.

This study was carried out in Slovakia in 22 different locations (Figure 1).

Directly after the sampling, all mushroom samples (n = 230) were cleaned up from any organic and inorganic debris and the bottom part of the stem was cut off. After that, they were divided into two parts: cap and stem. The individual cap and stem samples were sliced into pieces using a ceramic knife and dried to a constant weight at 45 °C in a laboratory dry heat-oven with forced air circulation (Memmert GmbH & Co. KG, Schwabach, Germany) for 22 h. The dried samples were pulverized in the rotary homogenizer (IKA Mills A 10 basic -Werke GmbH & Co. KG, Staufen, Germany) and stored in polyethylene bags until further analysis. Soil/substrate samples (n = 230) were collected together with mushroom samples at the same location from a depth of approximately 0.10 m. Under the laboratory conditions, the samples were air-dried at room temperature for 3 weeks. Afterward, they were sieved through a mesh sieve (2 mm) and stored in paper bags until the analysis.

### 2.3. Sample Analysis

Total mercury content was determined by cold-vapor AAS analyzer AMA 254 (Altec, Prague, Czech Republic), in all types of dried and homogenized samples (separately in 230 cap, stem, and soil/substrate samples (690 samples in total)). The limit of detection for Hg was set at 1.5 × 10^−6^ mg kg^−1^ DW and the limit of quantification at 4.45 × 10^−6^ mg kg^−1^ DW. Two Certified Reference Materials (CRM) from the Institute for Reference Materials and Measurements were used to check the accuracy and precision of the analytical method. The recovery value for the loam soil (ERM-CC141), varied between 0.06 and 0.07 mg kg^−1^ DW, and for the Mussel tissue (ERM-CE278k), it varied between 0.05 and 0.06 mg kg^−1^ DW.

### 2.4. Risk Assessment

To assess the level of ecological load of the monitored localities, we evaluated the Hg content in the soil/substrate using the following parameters:

Contamination factor ($C_{f}^{i}$) described by [20] was used to express the level of soil/substrate pollution by mercury. It is calculated as follows:

(1) $$C_{f}^{i}=\frac{C_{0-1}^{i}}{C_{n}^{i}}\;$$

where: $C_{0-1}^{i}$ is the total Hg content in soil and $C_{n}^{i}\;$ is the background Hg level, which is (0.06 mg kg^−1^) [21]. The contamination factor values are divided into four categories: low contamination factor ($C_{f}^{i}$ < 1); moderate contamination factor (1 $\leq C_{f}^{i}$ < 3); considerable contamination factor (3 $\leq C_{f}^{i}$ < 6); very high contamination factor ($C_{f}^{i}\;$≥ 6).

Ecological risk assessment is assessed by the potential ecological risk index (PER, or RI). PER expresses the amount of potential ecological risk factor for the given risk element ($E_{r}^{i}$) [20]:

(2) $${\mathrm{PER}}_{\mathrm{Hg}}=E_{r}^{i}$$

where: $E_{r}^{i}$ is the potential ecological risk factor of a single element (Hg). The degree of ecological risk can be categorized as follows: $E_{r}^{i}$ < 40: low risk; 40 ≤ $E_{r}^{i}$ < 80: moderate risk; 80 ≤ $E_{r}^{i}$ < 160: considerable risk; 160 ≤ $E_{r}^{i}$ < 320: high risk; $E_{r}^{i}$ ≥ 320: very high risk [22,23].

To quantify the level of contamination on the sampling localities the geo-accumulation index (I_geo_) was calculated:

I_geo =_
*log_2_* (*Cn*/1.5 × *Bi*) (3)

where: *Cn* is the Hg content in the soil samples, 1.5 is the constant that is used due to potential variations in the baseline data (characterizes the depositional feature, rock geology, and other effects) and *Bi* is the background value of Hg (0.06 mg kg^−1^) [21]. The I_geo_ values are divided into seven categories [24]: background values (I_geo_ ≤ 0); uncontaminated (0 < I_geo_ < 1); uncontaminated or slightly contaminated (1 ≤ I_geo_ < 2); slightly contaminated (2 ≤ I_geo_ < 3); moderately contaminated (3 ≤ I_geo_ < 4); strongly contaminated (4 ≤ I_geo_ < 5); very strongly contaminated (I_geo_ ≥ 5).

Bioaccumulation factor (BAF) was calculated to assess the level of transition and accumulation of Hg from soil/substrate to the above-ground parts (fruiting body) of *M. procera*. It was calculated as follows:

(4) $$\mathrm{BAF}=\frac{C_{Hg}}{C_{s}}$$

where: *C_Hg_* is the measured mercury content mushroom samples and *C_s_* is the measured mercury content in soil/substrate. BAF < 1 indicates excluders, BAF > 1 indicates accumulators [25]. The cap/stem quotient (Q_c/s_) was evaluated to compare the level of Hg translocation within the fruiting body.

### 2.5. Health Risk Assessment

#### Provisional Tolerable Weekly Intake—PTWI

The percentage of the provisional tolerable weekly intake (PTWI) assesses the health risk from exposure to each toxic element. The tolerable weekly intake per person weighing 70 kg for Hg is 0.28 mg per person per week [26]. Taking into account the average consumption of “Other vegetables and mushrooms” in Slovakia that was 0.23 kg FW per person per week in 2018 [27] the percentage of PTWI was calculated as follows:

(5) $$\mathrm{PTWI}\;\left(\%\right)=\frac{{\mathrm{BS}}_{\mathrm{HG}}\times 0.23}{0.28}\times 100$$

where: BS_Hg_ is the measured content of Hg in the biological sample (mg kg^−1^ of fresh weight (FW) in mushrooms). If the detected value was greater than 100%, the consumption of mushroom samples from the area is potentially hazardous.

### 2.6. Statistical Analysis

All statistical operations were performed using R studio version 1.2.5033 [28]. Spearman’s correlation analysis was used to determine the correlation relationship (negative or positive) between tested variables, soil/substrate, and mushroom, with a significant level *p* < 0.05 [29]. The Kruskal-Wallis test with multiple pairwise comparisons using the Wilcox test was used to identify significant differences between tested variables at the significance level of 5%, using the *p*-value.

## 3. Results and Discussion

### 3.1. Mercury in Soil/Substrate Samples

Soil/substrate represents the main source of nutrients as well as risk elements (e.g., Hg) for the mycelium and mushroom’s fruiting body. When compared with the fruiting bodies, the soil/substrate contains mercury in minor contents. Mercury content in soil/substrate and mushroom samples (stem and cap) is shown in Table 2.

Hodruša-Hámre, Jedľové Kostoľany, Krajné, Sitnianska lehôtka, Snina, Šachtička, Štiavnik and Zbyňov belong to the 1st environmental region quality (regions with unisturbed environment), while Bojná, Divina, Duchonka, Kráľovce, Limbach, Lozorno, Nemečky, Orovnica, Pezinská Baba, Pitelová, Skýcov, Solčany and Tesáre) belong to the 2nd environmental region quality (regions with moderately disturbed environment).

**Figure 2:**
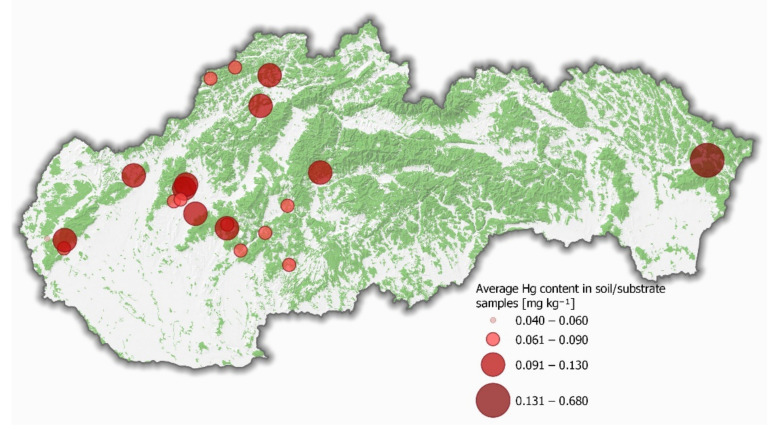
Distribution of mercury content in soil/substrate samples in the sampling localities.

In Slovakia, the background Hg content in the soil is 0.06 mg kg^−1^ [21]. The average mercury content in the soil/substrate in the study areas varied between 0.04 and 0.68 mg kg^−1^ DW (Figure 2). The highest average Hg content was detected in Snina (0.68 ± 0.19 mg kg^−1^ DW) and the lowest in Lozorno (0.04 ± 0.02 mg kg^−1^ DW). 

When comparing our obtained results with the background value for mercury in soil/substrates, only samples from Snina exceeded the limit. We assume that a higher content of mercury was recorded in this locality due to chemical industries and landfills for industrial and hazardous waste located nearby. Árvay et al. [30] studied mercury contents in edible wild-growing mushroom and soil/substrate samples (n = 33) collected from Central Slovakia. The content of total mercury in the soil/substrate varied between 0.05 and 0.27 mg kg^−1^ DW, while the highest average value of Hg content was detected in *Macrolepiota procera* (n = 8) and it was 0.13 mg kg^−1^. In another study, the content of total mercury in the underlying substrate ranged between 0.05 and 0.27 mg kg^−1^ (n = 33), while the average value of Hg content in the substrate in the case of mushroom samples with the highest content of mercury (*Macrolepiota procera* (Scop.) Singer, n = 5) was around 0.13 mg kg^−1^ ± 61.7 (mean ± RSD) ranging between 0.06 and 0.22 mg kg^−1^. The data of mercury content in the soil/substrate showed that none of the soil/substrate samples exceeded the mercury limit the soil/substrate [31]. Falandysz et al. [32] determined contents of mercury in the fruiting bodies of 15 species of higher mushrooms and soil/substrate collected from Wieluńska Upland in the northern part of Sandomierska Valley in south-central Poland. A total of 197 mushroom samples and 227 soil samples were analyzed. Mean mercury contents in soil substrate corresponding to 15 mushroom species (17 samples of *M. procera*) were between 0.03 ± 0.02 and 0.09 ± 0.06 mg kg^−1^ DW (total range between 0.03 and 0.19 mg kg^−1^ DW). According to Falandysz and Gucia [33], the range of mercury content in topsoil samples (Poland) was between 0.01 and 0.54 mg kg^−1^ DW (means ranged between 0.02 and 0.36 mg kg^−1^ DW). More recently, Mleczek [34] measured the mean mercury content 0.06 mg kg^−1^ DW (it ranged between 0.05 and 0.08) in the soil corresponding to *M. procera* from Polish forests.

### 3.2. Soil Pollution

To assess soil pollution, contamination factor, the index of geoaccumulation, and the potential ecological risk index were calculated for each sampling point. The values of C_f_, PER, and Igeo are shown in Table 2. The contamination factor for the sampling sites was determined as low (21 sampling sites), and moderate (1 sampling site). No locality was evaluated as considerable or very high contaminated. The highest value of mercury contamination factor was found in Pitelová (C_f_ = 2.86). The index of geoaccumulation was evaluated as uncontaminated for 5% of sampling sites, almost 73% as uncontaminated or slightly contaminated, 18% as slightly, and 5% as very strongly contaminated by Hg. The highest value of the geoaccumulation index of geoaccumulation value was found in Pitelová (I_geo_ = 11.4). Šefčík et al. [21] used the geoaccumulation index to study the contamination of Slovak soils. This study discovered that the most serious pollution was associated with mining activities and related industrial activities. The degree of the ecological risk of mercury was estimated as low risk for 1 sampling site (Kráľovce), moderate risk for 16 sampling sites, the considerable risk for 4 sampling sites, and very high risk for 1 sampling site. There was no locality where high risk was found. The potential ecological risk index was computed to detect the ecological risk of mercury in the analyzed soils/substrates. The majority of the studied soil samples showed moderate ecological risk (73%), while 18% of analyzed samples showed considerable ecological risk. Additionally, low (4.50%) and a very high ecological risk (4.5%) were confirmed. There was no locality where high ecological risk was found. The highest PER value was found in Pitelová (455). Based on these facts, we can conclude that the most mercury polluted soil was in Pitelová. These findings confirmed the fact that Pitelová belongs to the 2nd environmental quality region (region with the moderately disturbed environment) [35].

Kruskal-Wallis test was conducted to examine the differences in soil according to the sampling sites/localities. It has shown that there was a statistically significant difference between tested variables (*p* = 3 × 10^−14^). This test (Figure S1) showed that Lozorno is significantly lower, and Snina is significantly higher compared to all analyzed localities.

### 3.3. Mercury in M. procera Fruiting Bodies

The element accumulation by wild-growing mushrooms has been the subject of numerous scientific papers around the world. Depending on the collection site, a higher, lower, or significantly differentiated ability of mushrooms to accumulate some toxic elements were reported. However, the efficiency of the element accumulation does not always depend on their content in a soil/substrate, but the element contents in such cases depend on mushroom species, genus, or the families to which they belong [36]. In this line, Kalač [18] pointed out the hypothesis that the increasing age of mycelium, up to decades in wild-growing species, and a protracted interval between fructifications significantly elevate the contents of many elements in fruiting bodies and usually higher levels occur in caps than in stems. In the present study, the mercury content in mushroom stems varied from 0.03 to 2.00 mg kg^−1^ DW. The maximum content was detected in Pitelová (2.21 ± 0.26) and the minimum in Hodruša-Hámre (0.69 ± 0.36). Regarding mushroom caps, Hg content varied between 0.04 and 6.29 mg kg^−1^ DW. The highest recorded mercury average value was 4.14 ± 1.12 mg kg^−1^ DW (Pitelová) and the lowest 0.89 ± 0.30 mg kg^−1^ DW (Kráľovce). The threshold for Hg in edible mushrooms (both cap and stem) is 0.75 mg kg^−1^ FW. When compared with our detected mercury contents, all analyzed samples (both stems and caps) did not exceed the limit.

Árvay et al. [31] analyzed *M. procera* from Central Slovakia (Banská Bystrica) and they detected the average content of mercury in stems 1.40 (0.12–1.75) mg kg^−1^ DW. The highest mercury content was measured in *M. procera* cap, and the average value was 1.98 (between 0.41 and 3.20 mg kg^−1^ DW). Falandysz et al. [32] found out that Parasol Mushroom contained the greatest (compared with other species) mean mercury contents both in caps (between 4.50 ± 1.70 and 4.40 ± 2.40 mg kg^−1^ DW) and stems (between 2.80 ± 1.30 and 3.00 ± 2.00 mg kg^−1^ DW). The Parasol Mushroom was also characterized to have a great potential to bioaccumulate mercury from the soil as evidenced by great bioconcentration factors (BCFs), which was between 170 ± 160 and 130 ± 120 for caps, and 110 ± 97.0 and 89.0 ± 92.0 for stems. Moreover, Falandysz and Gucia [33] measured that the total mercury content ranged between 0.05 and 22.0 mg kg^−1^ DW and between 0.05 and 20.0 mg kg^−1^ DW in caps and stems of *M. procera*, respectively. The caps generally contained higher contents of mercury when compared to stems, and the cap to stem mercury content quotient ranged between 1.30 and 4.60. Mleczek et al. [36] determined 63 mineral elements in 17 wild-growing mushroom species from Wielkopolska Province in Poland. Detected mercury content in *M. procera* was 4.23 mg kg^−1^. The highest Hg contents were found in *M. procera* and S. bovinus. The common ranges reported in the literature for the elements of the trace elements with detrimental health effect are <0.5–5, <0.5–10, <0.2–10, <1–5, <0.5–5, and <1–5 mg kg^−1^ for Ag, As, Ba, Cd, Hg, and Pb, respectively. Mleczek et al. [33] studied 34 elements in four edible mushroom species: Boletus edulis, Imleria badia, Leccinum scabrum, and *Macrolepiota procera*, and associated soil collected from Polish forests between 1974 and 2019. The average detected mercury content in *M. procera* was 1.74 (between 0.62 and 2.80) mg kg^−1^ DW. The BCF values for *M. procera* ranged between 0.084 and 2.85 mg kg^−1^. A comparison of the content of the toxic metal was done and they found out that the selected species make a rather significant contribution to elements intake, and *M. procera* had a rather significant contribution to Hg intake. In general, our results (Table 2) show that *M. procera* proved its ability to accumulate Hg. Higher mercury levels were detected in caps than in stems. This fact verifies the hypothesis stated by Kalač [18], that usually higher levels occur in caps than in stems.

Kruskal-Wallis test has shown that there was a statistically significant difference between the tested variables (stem/locality), where *p* = 1.9 × 10^−11^. Statistical analysis (Figure S2) demonstrated that Kráľovce is significantly lower, and Pitelová is significantly higher compared to all studied localities. The relationship between cap and localities (Figure S3) showed that there is a statistically significant difference (*p* = 2 × 10^−14^) and Kráľovce is significantly lower, and Pitelová is significantly higher compared to all studied localities (same as for stems/localities).

The Kruskal-Wallis test with multiple pairwise comparisons was used to identify significant differences between tested variables (soil, stem, and cap samples) at the significance level of 5%, using the asymptotic *p*-value (Figure S4). This test showed that there was a statistically significant difference among all of the analyzed variables. It has also shown that the highest mercury contents were found in mushroom caps and the lowest in the soil/substrate. These findings also indicate mercury translocation from the soil/substrate to the mushroom fruiting body.

### 3.4. Bioaccumulation Factor (BAF) and Cap/Stem Quotient (Qc/s)

Mercury can be efficiently bioaccumulated by many mushrooms [37]. The bioaccumulation factor of *M. procera* ranged between 3.40 and 46.9 (caps) and 2.50 to 33.0 (stems). These results are closely correlated with the findings of other authors, who state that species with a BAF value > 1 accumulate the highest contents of risk elements in tubes and spores and flash of the cap [38]. In some studies, the BAF values of *M. procera* caps varied between 16 ± 6 and 220 ± 110 (total range between 0.52 and 470), and between 7.60 ± 2.60 and 130 ± 96 (total range between 0.52 and 340) for the stems [32,39]. The cap/stem quotient (Q_c/s_) was evaluated to compare the level of Hg translocation within the fruiting body. The quotient varied between 1.21 (Kráľovce) to 2.10 (Bojná). Falandysz et al. [39] indicate that the hymenophore of Parasol Mushroom is the morphological part, which is especially rich in mercury when compared to the rest of the fruiting body, and that the spores are probably characterized by elevated mercury accumulation in the hymenophore of this mushroom species.

### 3.5. Health Risk Assessment

In our study, %PTWI (using mean Hg content) ranged between 26% and 121% (for caps) and between 20.3 and 64.9% (for stems). The highest %PTWI for the cap was evaluated in Divina (105%) and Pitelová (121%). The recommended provisionally tolerable weekly intake from all of the analyzed samples was exceeded in 9% of the samples. Giannaccini et al. [40] analyzed caps and stems of 141 fruiting bodies of Parasol Mushroom and the surface layer of soils collected from 11 spatially distant areas in Northern Poland. They found out that the median mercury content in *M. procera* caps varied between 1.30 and 7.00 mg kg^−1^ DW. The estimated intake of Hg resulting from the consumption of 300 or 500 g portions of caps was assessed as 39–210 and 65–350 μg, and this is equivalent to 0.65–3.5 and 1.1–5.8 μg kg^−1^ body mass (BM) (a 76.0 kg adult). Taking into account that the provisionally tolerable weekly intake (PTWI) of mercury is 300 μg (equivalent to 5.00 or 0.70 μg kg^−1^ BM per day), and a reference dose of 0.30 μg kg^−1^ BM per day [41,42], mercury in *M. procera* caps for some areas might be of concern, especially if eaten by pickers frequently in the mushrooming season.

## 4. Conclusions

This study has been carried out to investigate the accumulation of mercury in edible wild-growing *M. procera* and its corresponding soil/substrate from 22 different localities in Slovakia. The Parasol Mushroom is a species that is harvested in the wild for its unique taste. All analyzed mushroom samples of *M. procera* did not exceed the limit for Hg in edible mushrooms (0.75 mg kg^−1^ FW), while recommended provisionally tolerable weekly intake was exceeded in 9% of the analyzed samples. The level of ecological load of the monitored localities showed that the analyzed soils/substrates were polluted with mercury. The potential of Parasol Mushrooms to bioaccumulate certain metals, e.g., mercury in fruiting bodies can be very or extremely high. The contamination factor for the sampling sites was determined as low and moderate. The degree of the ecological risk of mercury was estimated as low risk (1 sampling site), moderate risk (16 sampling sites), considerable risk (4 sampling sites), and very high risk (1 sampling site). The majority of the studied soil samples showed moderate ecological risk and considerable ecological risk. Moreover, low (4.50%) and very high ecological risks (4.5%) were confirmed. The highest value contamination factor, geoaccumulation index, and PER were determined in Pitelová. This reaffirms that Pitelová belongs to the regions with a moderately disturbed environment in Slovakia. The current study validates that *M. procera* can uptake and accumulate Hg (and other elements) from its underlying soil/ substrate. Information provided by our study can bring a more comprehensive understanding of mercury transmission from the soil/substrate to the mushroom fruiting bodies. Concurrently, it can fill the vacancy and provide information about a significant detrimental impact on the environment, as well as consumers’ health resulting from long-term consumption of contaminated mushrooms.

## Figures and Tables

**Table 1:** Basic characteristics of the sampling areas.

Locality	n	Coordinates (VGS 84)		Altitude (m)
Bojná	15	48.591916	18.021600	390
Divina	6	49.286883	18.691366	423
Duchonka	9	48.663350	18.094166	275
Hodruša-Hámre	21	48.468566	18.755716	409
Jedľové Kostoľany	9	48.495283	18.454450	420
Krajné	15	48.707103	17.688867	289
Kráľovce	20	48.326200	18.986500	229
Lazy pod Makytou	12	49.244400	18.225650	542
Limbach	7	48.294658	17.200095	420
Lozorno	8	48.335600	17.063083	220
Nemečky	6	48.683833	18.105333	298
Orovnica	8	48.366516	18.575166	254
Pezinská Baba	8	48.337583	17.200451	537
Pitelová	5	48.618097	18.913429	480
Sitnianska Lehôtka	20	48.312333	18.961416	232
Skýcov	10	48.476183	18.454166	473
Snina	7	48.976100	22.194766	260
Solčany	8	48.537475	18.198736	385
Šachtička	6	48.803309	19.150328	1009
Štiavnik	6	49.312900	18.412250	655
Tesáre	15	48.604333	18.073000	251
Zbyňov	9	49.124650	18.639466	378

**Table 2:** Mercury content in soil/substrate and mushroom samples and ecological and health risk indices.

Sampling Locality	Soil/Substrate				Mushroom						
	Hg (mg kg^−1^ DW) AVG ± S.D. Min–Max	Igeo *	Cf *	PER *	Hg_cap_ (mg kg^−1^ DW) AVG ± S.D. Min–Max	Hg_stem_ (mg kg^−1^ DW) AVG ± S.D. Min–Max	BAF_cap_ *	BAF_stem_ *	Q_c/s_ *	%PTWI_cap_ *	%PTWI_stem_ *
Bojná	0.09 ± 0.03 0.03–0.15	1.41	−0.20	56.5	2.09 ± 1.17 0.06–4.57	1.00 ± 0.46 0.05–1.55	24.7	11.7	2.10	61.3	29.2
Divina	0.13 ± 0.02 0.11–0.16	2.23	0.56	89.3	3.57 ± 1.66 1.84–6.29	1.75 ± 0.66 0.84–2.70	26.6	13.0	2.04	105	51.2
Duchonka	0.11 ± 0.05 0.05–0.18	1.79	0.12	71.6	1.89 ± 0.67 0.75–3.01	1.18 ± 0.29 0.65–1.61	17.6	10.9	1.61	55.3	34.5
Hodruša-Hámre	0.07 ± 0.03 0.03–0.14	1.10	−0.54	44.1	1.07 ± 0.60 0.07–2.86	0.69 ± 0.36 0.04–1.59	16.2	10.5	1.55	31.5	20.3
Jedľové Kostoľany	0.08 ± 0.03 0.04–0.11	1.31	−0.30	52.6	1.64 ± 1.49 0.77–5.75	0.91 ± 0.61 0.49–2.51	20.8	11.5	1.81	48.2	26.6
Krajné	0.10 ± 0.06 0.05–0.27	1.71	0.00	68.2	1.94 ± 0.49 1.16–2.95	1.31 ± 0.42 0.45–1.82	19.0	12.8	1.49	57.0	38.3
Kráľovce	0.06 ± 0.02 0.04–0.11	0.94	−0.73	37.5	0.89 ± 0.30 0.44–1.72	0.73 ± 0.23 0.44–1.59	15.7	13.0	1.21	26.0	21.5
Lazy pod Makytou	0.09 ± 0.02 0.06–0.13	1.53	−0.02	61.1	2.28 ± 0.87 1.22–4.00	1.46 ± 0.64 0.67–2.83	24.9	16.0	1.56	66.9	42.9
Limbach	0.07 ± 0.04 0.02–0.12	1.19	−0.61	47.5	2.73 ± 1.28 1.58–5.16	1.64 ± 0.60 0.92–2.70	38.3	23.1	1.66	80.0	48.2
Lozorno	0.04 ± 0.02 0.02–0.07	1.10	−0.74	43.8	1.59 ± 0.88 0.04–2.86	1.19 ± 0.69 0.03–2.17	43.9	33.0	1.33	46.5	34.9
Nemečky	0.11 ± 0.03 0.07–0.17	1.15	−0.38	46.1	1.03 ± 0.26 0.49–1.37	0.73 ± 0.16 0.58–1.04	9.76	6.95	1.40	30.1	21.5
Orovnica	0.07 ± 0.00 0.06–0.08	1.82	0.16	72.9	1.43 ± 0.41 0.95–2.15	0.79 ± 0.22 0.51–1.15	20.8	11.5	1.81	42.1	23.3
Pezinská Baba	0.12 ± 0.04 0.07–0.19	1.52	−0.12	60.9	2.81 ± 1.38 0.29–5.14	1.81 ± 0.71 0.24–2.64	22.9	14.7	1.56	82.5	53.0
Pitelová	0.09 ± 0.05 0.05–0.18	11.4	2.86	455	4.14 ± 1.12 2.32–5.74	2.21 ± 0.26 1.86–2.66	46.9	25.1	1.87	121	64.9
Sitnianska lehôtka	0.07 ± 0.03 0.04–0.14	2.23	0.46	89.2	1.08 ± 0.34 0.47–1.65	0.78 ± 0.27 0.31–1.28	15.4	11.0	1.40	31.8	22.7
Skýcov	0.13 ± 0.02 0.10–0.17	2.04	0.43	81.5	1.77 ± 0.74 0.86–3.40	0.91 ± 0.30 0.34–1.45	13.3	6.81	1.95	52.0	26.7
Snina	0.68 ± 0.19 0.37–0.89	1.56	−0.01	62.3	2.32 ± 0.37 1.73–2.89	1.70 ± 0.39 1.25–2.53	3.40	2.50	1.36	68.1	50.0
Solčany	0.13 ± 0.05 0.04–0.12	1.41	−0.20	56.5	2.34 ± 0.77 1.44–3.75	1.21 ± 0.38 0.78–1.88	17.5	9.04	1.94	68.7	35.5
Šachtička	0.12 ± 0.01 0.11–0.14	2.23	0.56	89.3	2.45 ± 0.13 2.23–2.65	1.74 ± 0.17 1.47–1.99	20.0	14.2	1.41	71.8	51.0
Štiavnik	0.08 ± 0.02 0.06–0.10	1.79	0.12	71.6	1.75 ± 0.75 0.98–3.05	0.97 ± 0.43 0.44–1.50	22.5	12.5	1.80	51.3	28.4
Tesáre	0.08 ± 0.02 0.05–0.11	1.10	−0.54	44.1	1.21 ± 0.28 0.81–1.66	0.88 ± 0.22 0.61–1.35	15.2	11.0	1.38	35.5	25.7
Zbyňov	0.13 ± 0.01 0.11–0.15	1.31	−0.30	52.6	1.93 ± 0.66 1.06–3.26	1.06 ± 0.47 0.07–1.76	15.5	8.52	1.81	56.6	31.2

* I_geo_: index of geoaccumulation, C_f_: contamination factor, PER: potential environmental risk, BAF: bioaccumulation factor, Q_c/s_: the cap/stem quotient, %PTWI: percentage of the provisional tolerable weekly intake.

## Data Availability

The datasets analyzed during the current study are available from the corresponding author on reasonable request.

## Supplementary Materials

The following are available online at https://www.mdpi.com/article/10.3390/jof7090772/s1, Figure S1: Mercury concentrations in soils/substrates, concerning localities. The horizontal line presents the average concentration for all samples, Figure S2: Mercury concentrations in mushroom stems, concerning localities. The horizontal line presents the average concentration for all samples, Figure S3: Mercury concentrations in mushroom caps, concerning localities. The horizontal line presents the average concentration for all samples, Figure S4: Mercury concentrations in soils/substrates and mushroom body parts (caps and stems).

## References

[1] Haro A., Trescastro A., Lara L., Fernández-Fígares I., Nieto R., Seiquer I. (2020). Mineral elements contents of wild growing edible mushrooms from the southeast of Spain. J. Food Compos. Anal..

[2] Kunca V., Pavlík M. (2019). Fruiting Body Production of, and Suitable Environmental Ranges for, Growing the Umbrella Polypore Medicinal Mushroom, *Polyporus umbellatus* (Agaricomycetes) in Natural Conditions in Central Europe. Int. J. Med. Mushrooms.

[3] Kunca V., Čiliak. M. (2017). Habitat preferences of *Hericium erinaceus* in Slovakia. Fungal Ecol..

[4] Árvay J., Demková L., Hauptvogl M., Michalko M., Bajčan D., Stanovič R., Tomáš J., Hrstková M., Trebichalský P. (2017). Assessment of Environmental and Health Risks in Former Polymetallic Ore Mining and Smelting Area, Slovakia: Spatial Distribution and Accumulation of Mercury in Four Different Ecosystems. Ecotoxicol. Environ. Saf..

[5] Melgar M.J., Alonso J., García M.A. (2016). Cadmium in edible mushrooms from NW Spain: Bioconcentration factors and consumer health implications. Food Chem. Toxicol..

[6] Bahadori M.B., Sarikurkcu C., Yalcin O.U., Cengiz M., Gungor H. (2019). Metal concentration, phenolics profiling, and antioxidant activity of two wild edible Melanoleuca mushrooms (*M. cognata* and *M. stridula*). Microchem. J..

[7] Mleczek M., Siwulski M., Stuper-Szablewska K., Rissmann I., Sobieralski K., Goliński P. (2013). Accumulation of elements by edible mushroom species: Part I. Problem of trace element toxicity in mushrooms. J. Environ. Sci. Health.

[8] Kokkoris V., Massas I., Polemis E., Koutrotsios G., Zervakis G.I. (2019). Accumulation of heavy metals by wild edible mushrooms with respect to soil substrates in the Athens metropolitan area (Greece). Sci. Total Environ..

[9] Melgar M.J., Alonso J., García M.A. (2009). Mercury in edible mushrooms and underlying soil: Bioconcentration factors and toxicological risk. Sci. Total Environ..

[10] Falandysz J., Chudzińska M., Barałkiewicz D.A.N.U.T.A., Saba M., Wang Y., Zhang J. (2017). Occurrence, variability and associations of trace metallic elements and arsenic in sclerotia of medicinal Wolfiporia extensa from polymetallic soils in Yunnan, China. Acta Pol. Pharm. Drug Res.

[11] Falandysz J., Hanć A., Barałkiewicz D., Zhang J., Treu R. (2020). Metallic and metalloid elements in various developmental stages of *Amanita muscaria* (L.) Lam. Fungal Biol..

[12] Falandysz J., Sapkota A., Dryżałowska A., Mędyk M., Feng X. (2017). Analysis of some metallic elements and metalloids composition and relationships in parasol mushroom *Macrolepiota procera*. Environ. Sci. Pollut. Res..

[13] Gucia M., Jarzyńska G., Rafał E., Roszak M., Kojta A.K., Osiej I., Falandysz J. (2012). Multivariate analysis of mineral constituents of edible Parasol Mushroom (*Macrolepiota procera*) and soils beneath fruiting bodies collected from Northern Poland. Environ. Sci. Pollut. Res..

[14] Vukojević V., Đurđić S., Stefanović V., Trifković J., Čakmak D., Perović V., Mutić J. (2019). Scandium, yttrium, and lanthanide contents in soil from Serbia and their accumulation in the mushroom *Macrolepiota procera* (Scop.) Singer. Environ. Sci. Pollut. Res..

[15] Hsu-Kim H., Eckley C.S., Achá D., Feng X., Gilmour C.C., Jonsson S., Mitchell C.P. (2018). Challenges and opportunities for managing aquatic mercury pollution in altered landscapes. Ambio.

[16] Xu J., Zhang J., Lv Y., Xu K., Lu S., Liu X., Yang Y. (2020). Effect of soil mercury pollution on ginger (Zingiber officinale Roscoe): Growth, product quality, health risks and silicon mitigation. Ecotoxicol. Environ. Saf..

[17] Tang W.L., Liu Y.R., Guan W.Y., Zhong H., Qu X.M., Zhang T. (2020). Understanding mercury methylation in the changing environment: Recent advances in assessing microbial methylators and mercury bioavailability. Sci. Total Environ..

[18] Kalač P. (2019). Mineral Composition and Radioactivity of Edible Mushrooms.

[19] Nayab G.U.L., Sardar K.H.A.N., Abbas K.H.A.N., Nawab J., Sarwar A., Nida G.U.L. (2020). Organic and Inorganic Mercury in Biological Samples of Flouresecent Lamp Industries Workers and Health Risks. Biomed. Environ. Sci..

[20] Hakanson L. (1980). An Ecological Risk Index for Aquatic Pollution Control. Water Res..

[21] Šefčík P., Pramuka S., Gluch A. (2008). Assessment of soil contamination in Slovakia according index of geoaccumulation. Agriculture.

[22] Islam S., Ahmed K., Masunaga S. (2015). Potential ecological risk of hazardous elements in different land-use urban soils of Bangladesh. Sci. Total Environ..

[23] Wu S., Peng S., Zhang X., Wu D., Luo W., Zhang T., Wu L. (2015). Levels and health risk assessments of heavy metals in urban soils in Dongguan, China. J. Geochem. Explor..

[24] Müller G. (1969). Index of geoaccumulation in sediments of the Rhine River. Geojournal.

[25] Dryźalowska A., Falandysz J. (2014). Bioconcentration of mercury by mushroom *Xerocomus chrysenteron* from the spatially distinct locations: Levels, possible intake and safety. Ecotoxicol. Environ. Saf..

[26] Joint F.A.O., World Health Organization, WHO Expert Committee on Food Additives (2012). Safety evaluation of certain food additives/prepared by the by the Seventy Fourth Meeting of the Joint FAO/WHO Expert Committee on Food Additives (JECFA). https://books.google.ro/books?hl=ro&lr=&id=YFAMU9qYD_YC&oi=fnd&pg=PP7&ots=e5skGh31Ln&sig=W99eNu8GUWHlWsG-ooJzM1dlkws&redir_esc=y#v=onepage&q&f=false.

[27] Statistical Organization of Slovak Republic 2019 Food Consumption in the Slovak Republic 2018. www.statistics.sk.

[28] RStudio Team (2020). RStudio: Integrated Development for R. RStudio.

[29] Addinsoft (2014). XLSTAT, Analyse de Données et Statistique avec MS Excel.

[30] Árvay J., Tomáš J., Hauptvogl M., Massányi P., Harangozo Ľ. (2015). Human Exposure to Heavy Metals and Possible Public Health Risks Via Consumption of Wild Edible Mushrooms from Slovak Paradise National Park, Slovakia. J. Environ. Sci. Health Part B.

[31] Árvay J., Záhorcová Z., Tomáš J., Hauptvogl M., Stanovič R., Harangozo Ľ. (2021). Mercury in edible wild-grown mushrooms from historical mining area–Slovakia: Bioaccumulation and risk assessment. J. Microbiol. Biotechnol. Food Sci..

[32] Falandysz J., Bielawski L., Kawano M., Brzostowski A., Chudzyński K. (2002). Mercury in mushrooms and soil from the Wieluńska Upland in south-central Poland. J. Environ. Sci. Health Part A.

[33] Falandysz J., Gucia M. (2008). Bioconcentration factors of mercury by Parasol Mushroom (*Macrolepiota procera*). Environ. Geochem. Health.

[34] Mleczek M., Siwulski M., Budka A., Mleczek P., Budzyńska S., Szostek M., Goliński P. (2021). Toxicological risks and nutritional value of wild edible mushroom species-a half-century monitoring study. Chemosphere.

[35] (2016). Environmental Regionalization of the Slovak Republic. https://www.minzp.sk/files/environmentalna-regionalizacia-sr.pdf.

[36] Mleczek M., Budka A., Kalač P., Siwulski M., Niedzielski P. (2020). Family and species as determinants modulating mineral composition of selected wild-growing mushroom species. Environ. Sci. Pollut. Res..

[37] Falandysz J., Borovička J. (2013). Macro and trace mineral constituents and radionuclides in mushrooms: Health benefits and risks. Appl. Microbiol. Biotechnol..

[38] Zocher A.L., Kraemer D., Merschel G., Bau M. (2018). Distribution of major and trace elements in the bolete mushroom *Suillus luteus* and the bioavailability of rare earth elements. Chem. Geol..

[39] Falandysz J., Gucia M., Mazur A. (2007). Content and bioconcentration factors of mercury by Parasol Mushroom Macrolepiota procera. J. Environ. Sci. Health Part B.

[40] Giannaccini G., Betti L., Palego L., Mascia G., Schmid L., Lanza M., Lucacchini A. (2012). The trace element content of top-soil and wild edible mushroom samples collected in Tuscany, Italy. Environ. Monit. Assess..

[41] World Health Organization (1978). Evaluation of Certain Food Additives and Contaminants. Twenty-Second Report of the Joint FAO/WHO Expert Committee on Food Additives.

[42] EPA, US (2005). Toxicological Review of Zinc and Compounds.

