# Supplementary material for: Mercury in Macrolepiota procera (Scop.) Singer and Its Underlying Substrate—Environmental and Health Risks Assessment
Source: J Fungi (Basel). 2021 Sep 18;7(9):772. doi: 10.3390/jof7090772 (PMC8467616; doi:10.3390/jof7090772)
Supplement: Supplementary file 1 [file jof-07-00772-s001.zip › jof-1326000-supplementary.pdf]

Supplementary Material

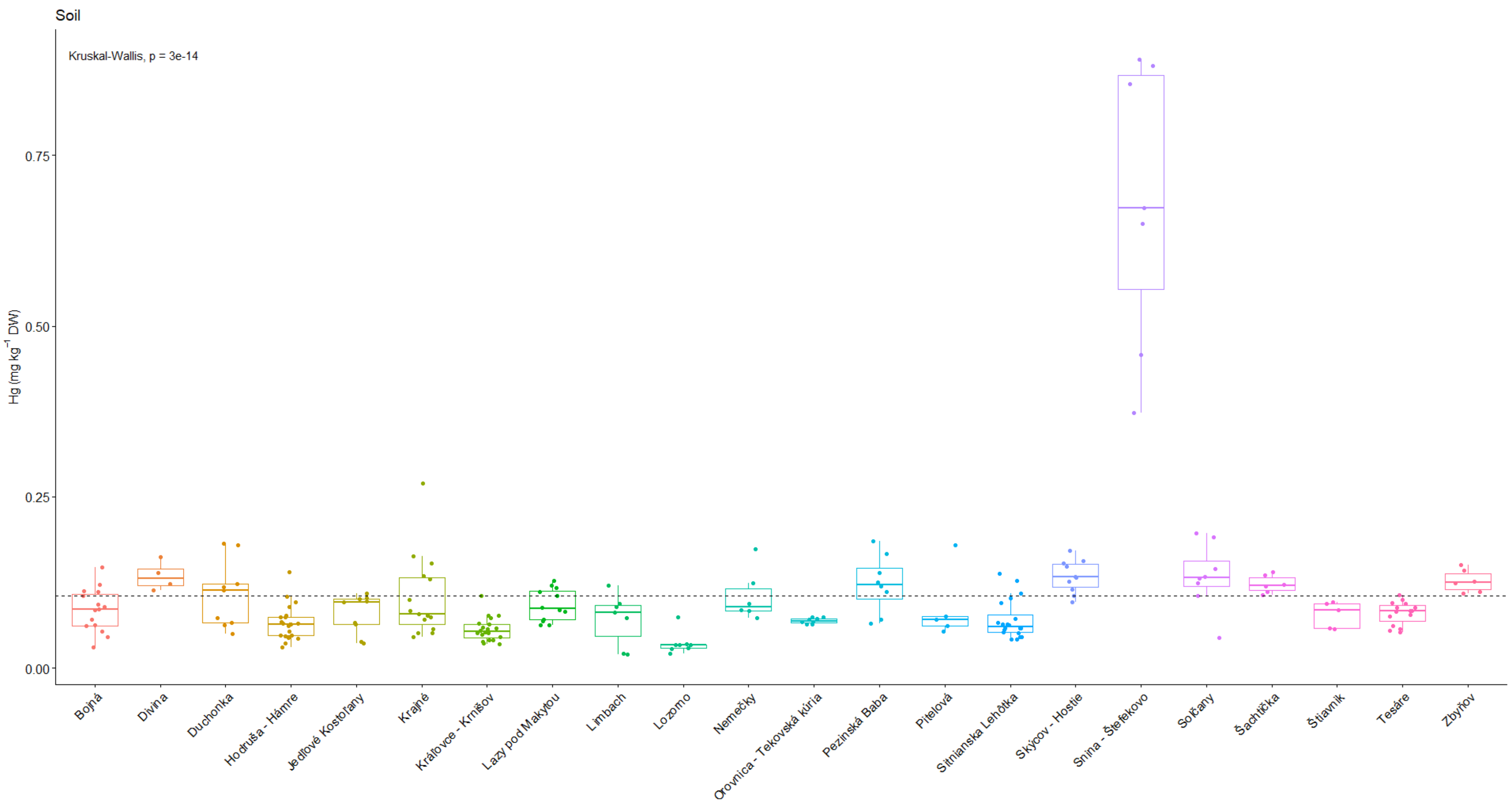

Figure S1. Mercury concentrations in soils/substrates, concerning localities. The horizontal line presents the average concentration for all samples.

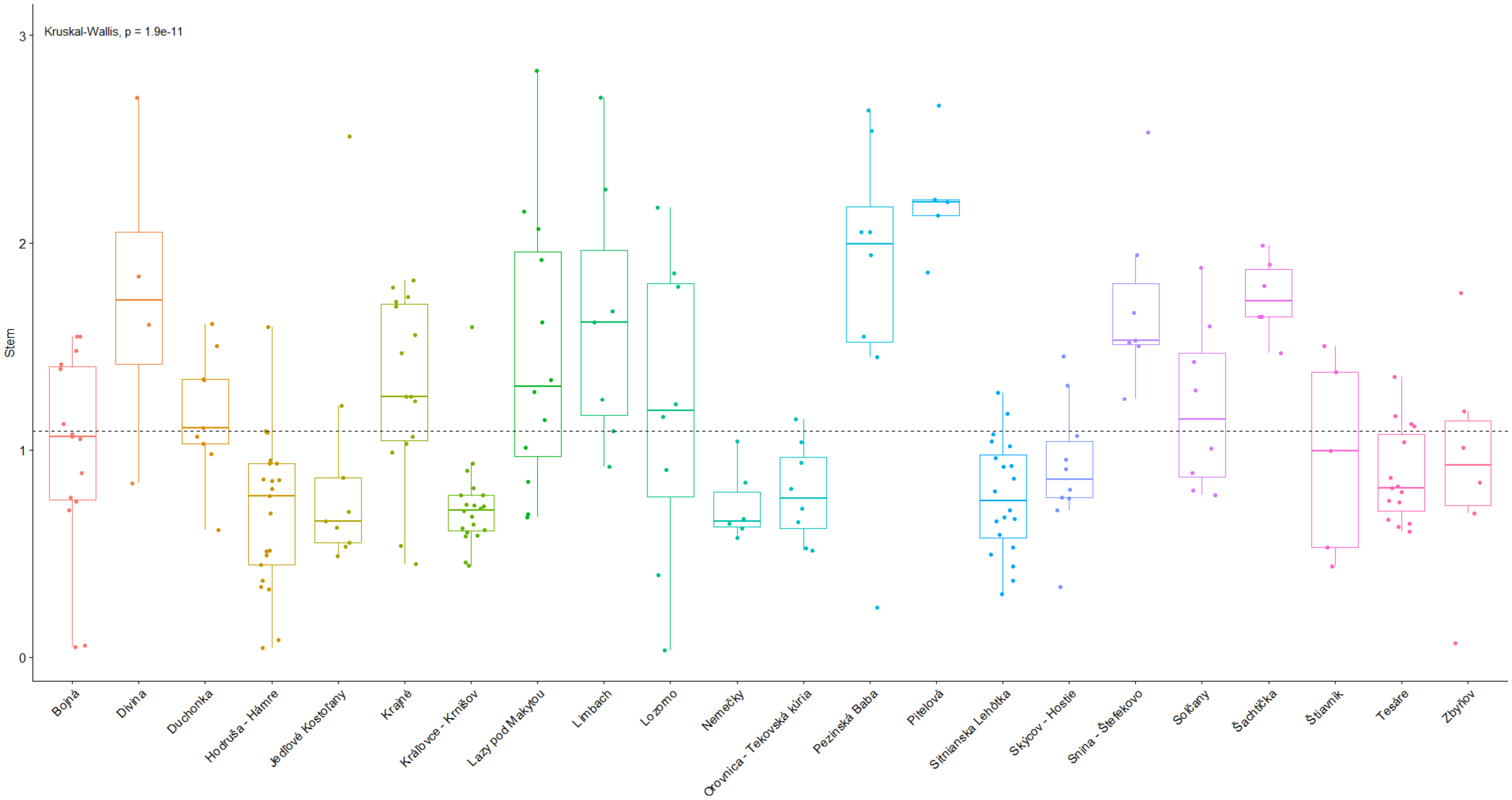

Figure S2. Mercury concentrations in mushroom stems, concerning localities. The horizontal line presents the average concentration for all samples.

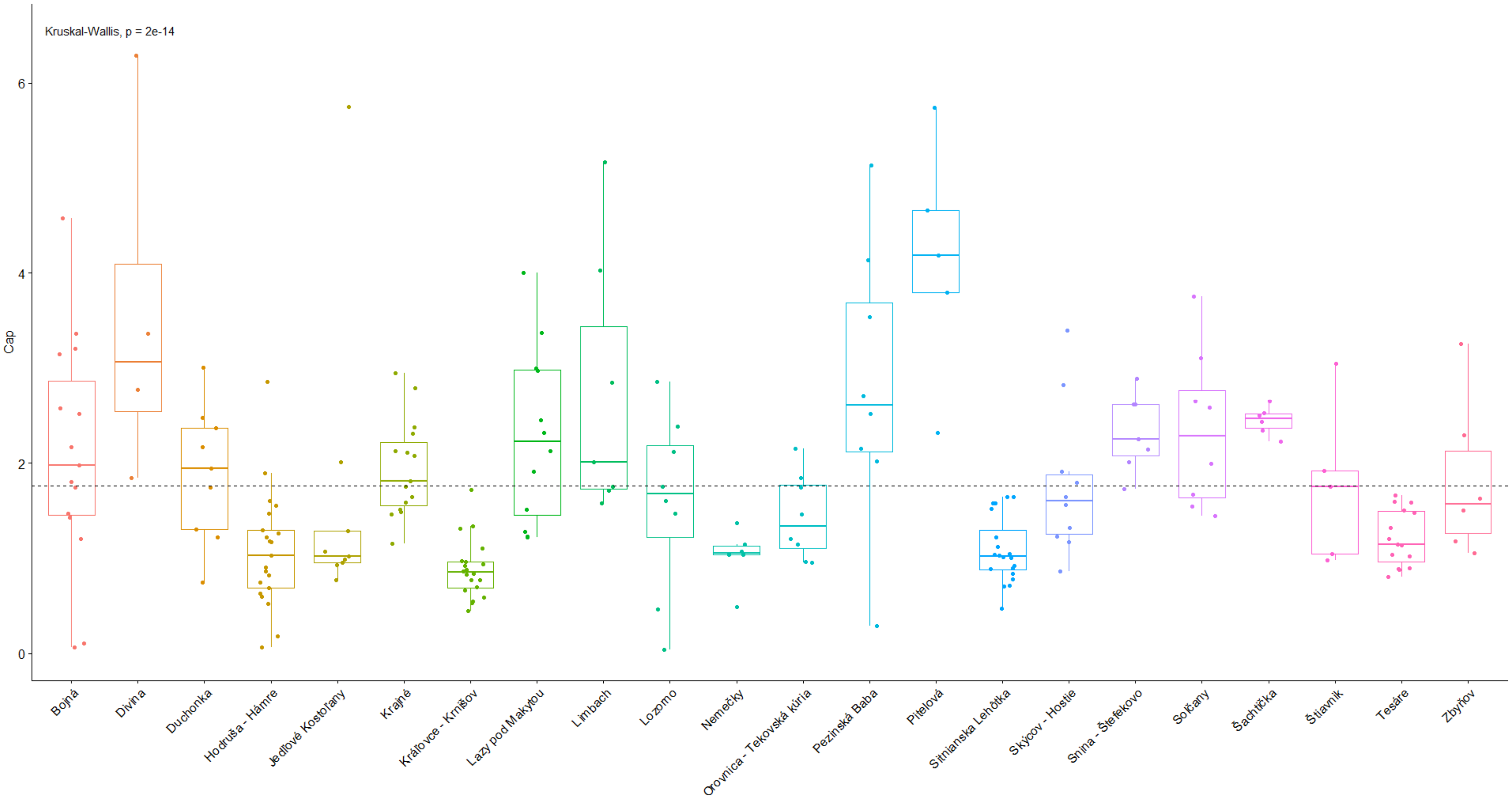

Figure S3. Mercury concentrations in mushroom caps, concerning localities. The horizontal line presents the average concentration for all samples.

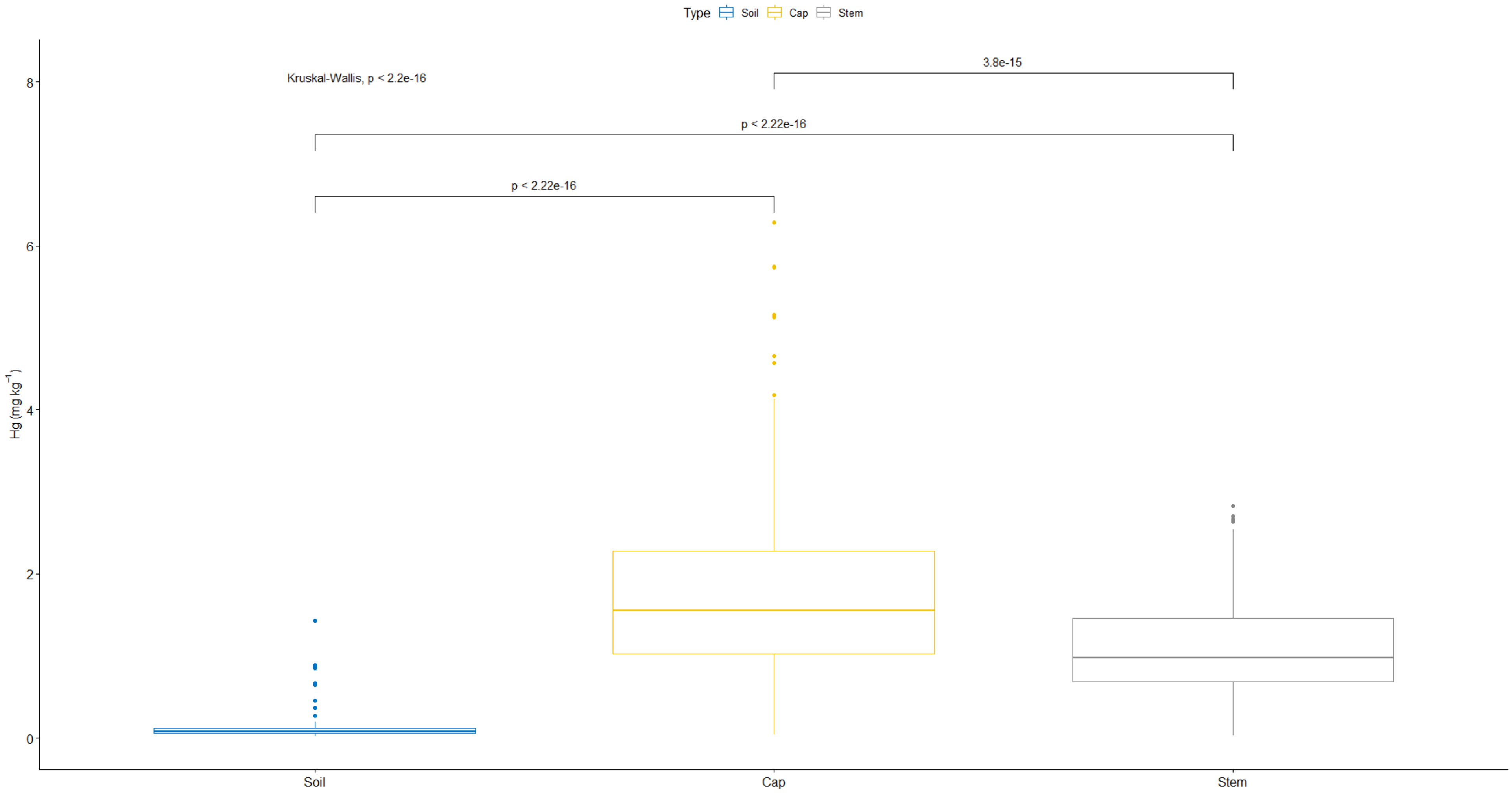

Figure S4. Mercury concentrations in soils/substrates and mushroom body parts (caps and stems).
